# Oral health practices and self-reported adverse effects of E-cigarette use among dental students in 11 countries: an online survey

**DOI:** 10.1186/s12903-022-02053-0

**Published:** 2022-01-26

**Authors:** Mohammed Nasser Alhajj, Sadeq Ali Al-Maweri, Morenike O. Folayan, Esam Halboub, Yousef Khader, Ridwaan Omar, Abdullah G. Amran, Ola B. Al-Batayneh, Asja Celebić, Sanja Persic, Humeyra Kocaelli, Firas Suleyman, Abdulaziz A. Alkheraif, Darshan D. Divakar, Abdulbaset A. Mufadhal, Mohammed A. Al-Wesabi, Wadhah A. Alhajj, Mokhtar A. Aldumaini, Saadika Khan, Thiyezen A. Al-Dhelai, Ahmed Shaher Alqahtani, Ali H. Murad, Joseph E. Makzoumé, Shivani Kohli, Tareq A. Ziyad

**Affiliations:** 1 Department of Prosthodontics, Faculty of Dentistry, Thamar University, Dhamar, Yemen; 2 College of Dental Medicine, QU Health, Qatar University, Doha, Qatar; 3 Department of Child Dental Health, Obafemi Awolowo University, Ile-Ife, Nigeria; 4 Department of Maxillofacial Surgery and Diagnostic Sciences, College of Dentistry, Jazan University, Jazan, Saudi Arabia; 5 Department of Oral Medicine, Oral Pathology and Oral Radiology, Faculty of Dentistry, Sana’a University, Sana’a, Yemen; 6 Department of Public Health, Community Medicine and Family Medicine, Faculty of Medicine, Jordan University of Science and Technology, Irbid, Jordan; 7 Department of Restorative Sciences, Division of Prosthodontics, Faculty of Dentistry, Kuwait University, Safat, Kuwait; 8 Department of Periodontics, Faculty of Dentistry, Thamar University, Dhamar, Yemen; 9 Department of Preventive Dentistry, Faculty of Dentistry, Jordan University of Science and Technology, Irbid, Jordan; 10 Department of Removable Prosthodontics, School of Dental Medicine and Clinical Hospital Centre, University of Zagreb, Zagreb, Croatia; 11 Department of Oral and Maxillofacial Surgery, Faculty of Dentistry, Istanbul University, Istanbul, Turkey; 12 Department of Prosthodontics, Faculty of Dentistry, University of Health Sciences, İIstanbul, Turkey; 13 Dental Biomaterials Research Chair, Dental Health Department, College of Applied Medical Sciences, King Saud University, Riyadh, Kingdom of Saudi Arabia; 14 Department of Conservative Dentistry, Faculty of Dentistry, Sana’a University, Sana’a, Yemen; 15 Department of Preventive and Biomedical Science, Faculty of Dentistry, University of Science and Technology, Taiz, Yemen; 16 Department of Periodontics, Faculty of Dentistry, Thamar University, Dhamar, Yemen; 17 Department of Dentistry, Faculty of Medical Sciences, Civilization University, Sana’a, Yemen; 18 Department of Fixed and Removable Prosthodontics, Faculty of Dentistry, University of Ibb, Ibb, Yemen; 19 Department of Restorative Dentistry, Faculty of Dentistry, University of the Western Cape, Cape Town, South Africa; 20 Department of Orthodontic and Pediatric Dentistry, College of Dentistry, Qassim University, Buraydah, Saudi Arabia; 21 Department of Orthodontics and Pediatric Dentistry, Faculty of Dentistry, University of Ibb, Ibb, Yemen; 22 Department of Maxillofacial Surgery and Diagnostic Sciences, College of Dentistry, Jazan University, Jazan, Saudi Arabia; 23 Department of Oral Diagnosis, College of Dentistry, University of Al-Qadisiyah, Al-Diwaniya, Iraq; 24 Department of Removable Prosthodontics, Faculty of Dental Medicine, Saint-Joseph University of Beirut, Beirut, Lebanon; 25 Division of Clinical Dentistry, School of Dentistry, International Medical University (IMU), Kuala Lumpur, Malaysia; 26 Private Dental Clinic, Sana’a, Yemen

**Keywords:** E-cigarette, Smoking, Dental students, Oral health, General health, Survey

## Abstract

**Objectives:**

E-cigarette use has become popular, particularly among the youth. Its use is associated with harmful general and oral health consequences. This survey aimed to assess self-reported oral hygiene practices, oral and general health events, and changes in physiological functions (including physical status, smell, taste, breathing, appetite, etc.) due to E-cigarette use among dental students.

**Methods:**

This online, multicounty survey involved undergraduate dental students from 20 dental schools across 11 different countries. The questionnaire included demographic characteristics, E-cigarette practices, self-reported complaints, and associated physiological changes due to E-cigarette smoking. Data were descriptively presented as frequencies and percentages. A Chi-square test was used to assess the potential associations between the study group and sub-groups with the different factors. Statistical analysis was performed using SPSS at *P* < 0.05.

**Results:**

Most respondents reported regular brushing of their teeth, whereas only 70% used additional oral hygiene aids. Reported frequencies of complaints ranged from as low as 3.3% for tongue inflammation to as high as 53.3% for headache, with significant differences between E-cigarette users and non-users. Compared to non-smokers, E-cigarette users reported significantly higher prevalence of dry mouth (33.1% vs. 23.4%; *P* < 0.001), black tongue (5.9% vs. 2.8%; *P* = 0.002), and heart palpitation (26.3%% vs. 22.8%; *P* = 0.001). Although two-thirds of the sample reported no change in their physiological functions, E-cigarette users reported significant improvement in their physiological functions compared to never smokers or tobacco users.

**Conclusion:**

Dental students showed good oral hygiene practices, but E-cigarette users showed a higher prevalence of health complications.

## Introduction

Electronic cigarettes (E-cigarette) or electronic nicotine delivery systems are a relatively new phenomenon amongst tobacco smokers largely because of claims that they may help with smoking cessation [1, 2]. E-cigarette use is increasingly popular among the youth and young adults [3]. Indeed, it has been the most popular form of smoking among the youth since 2014 [4, 5]. Typically, E-cigarette users inhale an aerosol containing nicotine, flavorings, and other additives [1]. The Food and Drug Administration (FDA), however, has reported that E-cigarette cartridges and solutions contain contaminants that are potentially harmful to humans: at a minimum, these contaminants cause irritation and inflammation of the airway epithelium [6, 7] and suppressed the immune response of the nasal mucosa [8]. In fact, the proinflammatory signals, and immune-suppressive changes in the respiratory mucosa of E-cigarette uses have been reported to be different from those of non-smokers [8, 9]. Recently, a United States court has recognized the E-cigarette as a smoking tobacco product that should be prohibited or regulated as a dangerous nicotine delivery system that needs to comply with the Federal Food Drug and Cosmetic Act [10].

As with traditional cigarettes, E-cigarettes have oral health consequences [9, 11, 12]. Basically, the oral cavity, being the first part of the body exposed to the constituents of E-cigarettes or any other forms of tobacco, is at increased risk of exposure to the carcinogenic, immunologic, microbial, and clinical effects of these products. The viscosity of e-liquid promotes the colonization of Streptococcus mutans, a major causative factor of dental caries [9]. It has been found that the major ingredients of the e-liquid (nicotine, acetaldehyde, acrolein, formaldehyde and flavoring chemicals like cinnamaldehyde [13, 14]) modify the oral microbiome toward higher abundance of oral pathobionts, alter host response, and enhance periodontal inflammation [11]. Nicotine, a well-known vasoconstrictor, decreases gingival blood flow, and depresses cytokine production (IL-1β, IL-2, TNF-α, and IFN-γ), neutrophil number, and immune cell function, leading ultimately to periodontal disease and tooth loss [15–17]. The propylene glycol content of the E-cigarette breaks down into acetic and lactic acids, and other harmful compounds to the enamel and oral soft tissue [18]. In addition, E-cigarette use can lead to hyposalivation resulting in tissue drying that promotes caries, periodontal disease, and other oral health problems [9, 11, 12, 19]. The vegetable glycerin with other flavoring agents can increase microbial adhesion to enamel and promote biofilm formation, which results in a decreased enamel hardness [18]. Cinnamaldehyde can suppress the phagocytic function of neutrophils and macrophages; and inhibit the natural killer cells’ cell–killing ability of tumor cells, and ciliary beating of airway epithelial cells [20, 21].

Good oral health behaviors may lessen the negative impact of E-cigarette use on oral health; and, of course, quitting smoking is the most effective way to ensure improved oral health [22–24]. Toothbrushing twice a day with fluoride-containing toothpaste, along with adjunct mouth rinses, should help reduce the risk of caries and periodontal disease [25–27]. Additionally, reducing the daily frequency of refined carbohydrate intake can reduce the risk of caries [28], and regular dental visits will facilitate prompt detection of oral lesions and the institution of caries reversal protocols.

Dental health professionals report their own oral health status more reliably, and are clearly more aware of the positive impact of good oral health behaviors on oral health than the general public [26]. There has always been a strong drive for active engagement of dental practitioners in smoking cessation programs [29–31]. Therefore, dental professionals would be a good study cohort to assess the relationship between oral health practices and cigarette smoking. Unfortunately, only a few studies have assessed dental students’ attitudes and knowledge about the negative effects of E-cigarette use [32, 33]. Hence, there is a paucity of information related to reported health effects, and negative impact of E-cigarette on oral health, particularly among health sciences students. In this study, we aimed to explore the self-reported practices of oral healthcare and hygiene measures, and the possible adverse effects of E-cigarette among dental students in different countries.

## Materials and methods

This study is a part of a large multinational survey conducted on E-cigarette use amongst dental students. An online cross-sectional survey was conducted among undergraduate dental students from 20 dental schools in 11 countries. Postgraduate students, academic staff, assistants, or technicians were excluded. The study was approved primarily by the Research Ethics Committee at the Faculty of Dentistry, Thamar University (Ref #2019003). In parallel with that, ethical clearance was obtained from all respective participating universities. The study fully complied with the Helsinki declaration. The electronic questionnaire was distributed to the target students via different methods including social media such as WhatsApp and Facebook groups. A hard copy of the questionnaire was also available for coauthors who could meet the dental students face-to-face. The survey had an introductory statement about the study team, study objectives, and confidentiality assurance. Each participant was able to answer the survey once and to edit their answers freely until they chose to submit. By clicking submit it was considered that the student consented to participate in the study.

The questionnaire comprised close-ended questions adopted from some previous studies [34–37] and covered: (1) sociodemographic data (age, sex, current educational level); (2) tobacco and E-cigarette use (current habits, frequency, etc.); (3) self-perceived oral health, including number of decayed, filled, and missing teeth (DMFT); (4) oral hygiene practices, including frequency of brushing, additional aids, type of toothpaste, dental visits, etc.; (5) self-perceived symptoms due to smoking; and (6) self-perceived changes in physiological functions (including physical status, smell, taste, breathing, appetite, etc.).

### Statistical analysis

Responses were exported to Excel sheets (MS Excel 2016) where they were checked, coded, and then transferred to a statistical software program (SPSS V25, IBM Corp. USA). Descriptive statistics in terms of frequencies and percentages were obtained for the study variables according to the smoking status (non-smokers, tobacco smokers, E-cigarette users, and dual users). The potential associations between the outcome and explanatory variables were assessed by Chi-square test, with a P-value less than 0.5 considered statistically significant.

## Results

A total of 5697 dental students from 11 different countries (Croatia, Iraq, Jordan, Kuwait, Lebanon, Malaysia, Nigeria, Saudi Arabia, South Africa, Turkey, and Yemen) took part in the study. Numbers varied greatly across countries, ranging from 110 in Kuwait to 1626 in Yemen. Of the total participants, 51.1% (n = 2909) were in the clinical years of study. The majority were females (60.5%, n = 3433) and unmarried (94.1%, n = 5361) (Table 1).

**Table 1:** Characteristics of participants by country (N = 5697); % in brackets

	Total	Gender		Age groups		Educational level		Marital status	
		Male	Female	≤ 20 years	> 20 years	Pre-clinical	Clinical	Married	Unmarried
All	5697 (100.0)	2264 (39.7)	3433 (60.3)	1844 (32.4)	3853 (67.6)	2788 (48.9)	2909 (51.1)	336 (5.9)	5361 (94.1)
Croatia	233 (4.1)	43 (18.5)	190 (81.5)	64 (27.5)	169 (72.5)	109 (46.8)	124 (53.2)	5 (2.1)	228 (97.9)
Iraq	369 (6.5)	129 (35.0)	240 (65.0)	146 (39.6)	223 (60.4)	227 (61.5)	142 (38.5)	11 (3.0)	358 (97.0)
Jordan	461 (8.1)	123 (26.7)	338 (73.3)	205 (44.5)	256 (55.5)	303 (65.7)	158 (34.3)	6 (1.3)	455 (98.7)
Kuwait	110 (1.9)	12 (10.9)	98 (89.1)	25 (22.7)	85 (77.3)	39 (35.5)	71 (64.5)	4 (3.6)	106 (96.4)
Lebanon	257 (4.5)	82 (31.9)	175 (68.1)	137 (53.3)	120 (46.7)	118 (45.9)	139 (54.1)	1 (0.4)	256 (99.6)
Malaysia	148 (2.6)	35 (23.6)	113 (76.4)	38 (25.7)	110 (74.3)	32 (21.6)	116 (78.4)	0 (0.0)	148 (100.0)
Nigeria	240 (4.2)	138 (57.5)	102 (42.5)	49 (20.4)	191 (79.6)	64 (26.7)	176 (73.3)	10 (4.2)	230 (95.8)
Saudi Arabia	596 (10.5)	292 (49.0)	304 (51.0)	91 (15.3)	505 (84.7)	178 (29.9)	418 (70.1)	55 (9.2)	541 (90.8)
South Africa	204 (3.6)	60 (29.4)	144 (70.6)	97 (47.5)	107 (52.5)	81 (39.7)	123 (60.3)	7 (3.4)	197 (96.6)
Turkey	1453 (25.5)	695 (47.8)	758 (52.2)	640 (44.0)	813 (56.0)	710 (48.9)	743 (51.1)	33 (2.3)	1420 (97.7)
Yemen	1626 (28.5)	655 (40.3)	971 (59.7)	352 (21.6)	1274 (78.4)	927 (57.0)	699 (43.0)	204 (12.5)	1422 (87.5)

As presented in Table 2, 1112 (19.6%) students were current smokers in the following proportions: 596 (10.5%) were tobacco smokers, 255 (4.5%) were E-cigarette users, and 261 (4.6%) were tobacco/E-cigarette dual users. The distribution of respondents by their current smoking status differed significantly by sex and country of origin. With regard to sex, there were significantly more males than females who smoke tobacco, E-cigarettes, or both. With regard to country, Nigeria had the highest proportion of respondents who had never smoked; Saudi Arabia had the least proportion of respondents who had never smoked; no respondent smoked tobacco in Kuwait and no respondent was a dual smoker in Nigeria. No statistically significant associations were observed between smoking status and marital status, age, and education level.

**Table 2:** Patterns of E-cigarette use or tobacco cigarette smoking by different grouping factors (N = 4564); % in brackets

	Total	Smoking status				*P*
		Never smoke	Tobacco only	E-cig. only	Dual user	
All	5676 (100.0)	4564 (80.4)	596 (10.5)	255 (4.5)	261 (4.6)	
Country						< 0.001
Croatia	233 (4.1)	175 (75.1)	49 (21.0)	5 (2.1)	4 (1.7)	
Iraq	369 (6.5)	300 (81.3)	31 (8.4)	11 (3.0)	27 (7.3)	
Jordan	461 (8.1)	343 (74.4)	45 (9.8)	44 (9.5)	29 (6.3)	
Kuwait	110 (1.9)	103 (93.6)	0 (0.0)	7 (6.4)	0 (0.0)	
Lebanon	257 (4.5)	220 (85.6)	27 (10.5)	4 (1.6)	6 (2.3)	
Malaysia	148 (2.6)	139 (93.9)	1 (0.7)	3 (2.0)	5 (3.4)	
Nigeria	240 (4.2)	228 (95.0)	7 (2.9)	5 (2.1)	0 (0.0)	
Saudi Arabia	592 (10.4)	430 (72.6)	55 (9.3)	69 (11.7)	38 (6.4)	
South Africa	204 (3.6)	177 (86.8)	16 (7.8)	7 (3.4)	4 (2.0)	
Turkey	1453 (25.6)	1090 (75.0)	208 (14.3)	59 (4.1)	96 (6.6)	
Yemen	1609 (28.3)	1359 (84.5)	157 (9.8)	41 (2.5)	52 (3.2)	
Gender						< 0.001
Male	2258 (39.8)	1594 (70.6)	344 (15.2)	176 (7.8)	144 (6.4)	
Female	3418 (60.2)	2970 (86.9)	252 (7.4)	79 (2.3)	117 (3.4)	
Age group						0.227
≤ 20 years	1838 (32.4)	1502 (81.7)	178 (9.7)	72 (3.9)	86 (4.7)	
> 20 years	3838 (67.6)	3062 (79.8)	418 (10.9)	183 (4.8)	175 (4.6)	
Marital status						0.090
Married	336 (5.9)	259 (77.1)	40 (11.9)	13 (3.9)	24 (7.1)	
Unmarried	5340 (94.1)	4305 (80.6)	556 (10.4)	242 (4.5)	237 (4.4)	
Educational level					0.076	
Pre-clinical	2776 (48.9)	2242 (80.8)	295 (10.6)	105 (3.8)	134 (4.8)	
Clinical	2900 (51.1)	2322 (80.1)	301 (10.4)	150 (5.2)	127 (4.4)	

The reported DMFT and oral hygiene practices are presented in Table 3. Slightly more than one-third of the sample (35.8%, n = 2029) reported having DMFT ≥ 3, 30.9% (n = 1748) reported to having DMFT < 3, and 33% (n = 1889) reported having DMFT = 0, with no statistically significant differences according to the students’ smoking status. A majority of students reported brushing their teeth 2 times or more daily (63.8%) and using fluoride-containing toothpaste (65.6%), with significant differences according to their smoking status (*P* = 0.003 each). Almost 80% of the participants reported that they eat sweets or drink sugary soft drinks on a daily (36.7%) or weekly basis (45.3%), and up to 72% of the participants reported that they use other oral-care devices, but with no significant differences according to the smoking status (*P* = 0.104 and *P* = 0.217, respectively). Most participants (n = 3069, 54.4%) reported regularly visiting the dentist (at least once a year), with a significant difference between E-cigarette users and other groups: 66.1% of E-cigarette smokers compared to 54.3% of non-smokers, 49.3% of tobacco smokers (49.3%), and 55.9% of dual smokers.

**Table 3:** DMFT and oral hygiene practices among dental students based on the current status of smoking

	Total	Smoking status				*P*
		Never smoke	Tobacco only	E-cigarette only	Dual user	
Number of decayed, filled, and missing teeth do you have?						0.517
None	1889 (33.3)	1525 (33.5)	198 (33.3)	77 (30.2)	89 (34.1)	
< 3 teeth	1748 (30.9)	1403 (30.8)	171 (28.7)	92 (36.1)	82 (31.4)	
≥ 3 teeth	2029 (35.8)	1627 (35.7)	226 (38.0)	86 (33.7)	90 (34.5)	
How many times do you brush your teeth per day?						0.003
None	255 (4.5)	185 (4.1)	44 (7.4)	16 (6.3)	10 (3.8)	
Once a day	1799 (31.7)	1449 (31.8)	195 (32.8)	83 (32.5)	72 (27.6)	
≥ 2 times a day	3614 (63.8)	2924 (64.2)	355 (59.8)	156 (61.2)	179 (68.6)	
Do you use fluoride containing toothpaste?						0.003
Yes	3709 (65.6)	3013 (66.3)	356 (59.9)	181 (71.3)	159 (61.2)	
No	608 (10.8)	476 (10.5)	85 (14.3)	21 (8.3)	26 (10.0)	
I don't know	1334 (23.6)	1054 (23.2)	153 (25.8)	52 (20.5)	75 (28.8)	
How often do you eat sweets or drink sugary soft drinks?						0.104
On a daily basis	2081 (36.7)	1698 (37.3)	217 (36.5)	92 (36.1)	74 (28.4)	
On a weekly basis	2569 (45.3)	2041 (44.8)	279 (46.9)	120 (47.1)	129 (49.4)	
Rarely	1016 (17.9)	816 (17.9)	99 (16.6)	43 (16.9)	58 (22.2)	
Do you use other oral-care devices besides toothbrush and toothpaste?						0.217
No	1594 (28.2)	1291 (28.4)	176 (29.7)	59 (23.2)	68 (26.1)	
Yes	4056 (71.8)	3251 (71.6)	417 (70.3)	195 (76.8)	193 (73.9)	
How often do you visit your dentist?						0.002
> 2 times a year	737 (13.1)	585 (12.9)	71 (12.0)	39 (15.4)	42 (16.1)	
1–2 times a year	2332 (41.3)	1880 (41.4)	221 (37.3)	127 (50.0)	104 (39.8)	
Rarely	2577 (45.6)	2073 (45.7)	301 (50.8)	88 (34.6)	115 (44.1)	

Table 4 shows the subjective complaints reported by respondents. The frequencies of perceived health problems associated with smoking ranged from as few as 3.3% who reported tongue inflammation to as many as 53.3% who reported headache. Compared to non-smokers, tobacco users, E-cigarette users, and dual users reported significantly higher prevalence of dry mouth (29.3%, 33.1% and 28.1%, respectively, vs. 23.4%; *P* < 0.001), black tongue (3.9%, 5.6% and 6.1%, respectively, vs 2.8%; *P* = 0.002), and heart palpitation (29.6%, 26.3% and 28.4%, respectively, vs. 22.8%; *P* = 0.001).

**Table 4:** Perceived related effects/events among dental students based on the current status of smoking

	Total	Smoking status				*P*
		Never smoke	Tobacco only	E-cigarette only	Dual user	
Have you experienced the following problems in the last month?						
Sore mouth and/or throat	1600 (28.3)	1314 (28.9)	162 (27.4)	54 (21.4)	70 (26.8)	0.062
Dry mouth and/or throat	1367 (24.7)	1042 (23.4)	172 (29.3)	81 (33.3)	72 (28.1)	< 0.001
Mouth and/or tongue inflammation	517 (9.2)	425 (9.4)	52 (8.8)	25 (9.9)	15 (5.7)	0.245
Black tongue	182 (3.2)	129 (2.8)	23 (3.9)	14 (5.6)	16 (6.1)	0.002
Gingivitis	1077 (19.1)	883 (19.4)	111 (18.7)	42 (16.7)	41 (15.8)	0.354
Nose bleeding	485 (8.6)	390 (8.6)	46 (7.7)	21 (8.4)	28 (10.7)	0.557
Headache	3015 (53.3)	2476 (54.5)	311 (52.4)	112 (44.3)	116 (44.6)	< 0.001
Cough	1635 (28.9)	1293 (28.5)	198 (33.4)	62 (24.6)	82 (31.4)	0.026
Chest pain	1000 (17.7)	756 (16.7)	141 (23.7)	44 (17.5)	59 (22.7)	< 0.001
Dizziness	1715 (30.7)	1390 (30.9)	179 (30.4)	68 (27.4)	78 (30.1)	0.698
Heart palpitation	1351 (23.9)	1035 (22.8)	176 (29.6)	66 (26.3)	74 (28.4)	0.001
Allergy	997 (17.7)	823 (18.1)	93 (15.7)	35 (13.9)	46 (17.6)	0.195

Yes response is reported

Table 5 highlights the self-reported changes in physiological functions within the past month as reported by respondents. Overall, a majority reported “no change” in their physiological functions (61.3–90.0%). Specifically, most E-cigarette users (range 66.3–81%) reported no change in their physiological functions. Surprisingly, significantly higher percentages of E-cigarette users reported “improvement” in most of their physiological functions compared to non-smokers, tobacco users and dual users (*P* < 0.05).

**Table 5:** Perceived physiological functions among dental students based on the current status of smoking

	Total	Smoking status				*P*
		Never smoke	Tobacco only	E-cigarette only	Dual user	
*Have you experienced changes in the following physiological functions in the past month?*						
Physical status						< 0.001
Worsened	564 (10.3)	436 (10.0)	89 (15.1)	13 (5.2)	26 (10.2)	
No change	4362 (79.8)	3520 (80.6)	454 (76.8)	193 (76.6)	195 (76.2)	
Improved	542 (9.9)	413 (9.5)	48 (8.1)	46 (18.3)	35 (13.7)	
Smell						< 0.001
Worsened	259 (4.7)	175 (4.0)	64 (10.8)	9 (3.6)	11 (4.3)	
No change	4873 (89.1)	3958 (90.6)	497 (84.2)	202 (80.2)	216 (84.4)	
Improved	335 (6.1)	236 (5.4)	29 (4.9)	41 (16.3)	29 (11.3)	
Taste						< 0.001
Worsened	224 (4.1)	148 (3.4)	53 (9.0)	11 (4.4)	12 (4.7)	
No change	4918 (90.0)	3998 (91.5)	499 (84.4)	204 (81.0)	217 (84.8)	
Improved	325 (5.9)	222 (5.1)	39 (6.6)	37 (14.7)	27 (10.5)	
Breathing						< 0.001
Worsened	546 (10.0)	376 (8.6)	106 (17.9)	25 (10.0)	39 (15.2)	
No change	4564 (83.5)	3742 (85.7)	442 (74.8)	185 (74.0)	195 (76.2)	
Improved	354 (6.5)	249 (5.7)	43 (7.3)	40 (16.0)	22 (8.6)	
Appetite						0.030
Worsened	869 (16.0)	688 (15.8)	115 (19.5)	30 (12.0)	36 (14.1)	
No change	3879 (71.2)	3114 (71.6)	407 (69.0)	175 (70.3)	183 (71.8)	
Improved	697 (12.8)	549 (12.6)	68 (11.5)	44 (17.7)	36 (14.1)	
Mood						< 0.001
Worsened	1437 (26.3)	1179 (27.0)	172 (29.1)	30 (11.9)	56 (21.9)	
No change	3355 (61.3)	2682 (61.4)	349 (59.0)	167 (66.3)	157 (61.3)	
Improved	678 (12.4)	509 (11.6)	71 (12.0)	55 (21.8)	43 (16.8)	
Memory						< 0.001
Worsened	907 (16.6)	743 (17.0)	110 (18.6)	18 (7.2)	36 (14.1)	
No change	4087 (74.8)	3263 (74.8)	430 (72.8)	198 (78.9)	196 (76.6)	
Improved	469 (8.6)	359 (8.2)	51 (8.6)	35 (13.9)	24 (9.4)	
Quality of sleep					0.003	
Worsened	1257 (23.0)	1015 (23.2)	157 (26.6)	35 (13.9)	50 (19.5)	
No change	3441 (62.9)	2750 (62.9)	355 (60.1)	175 (69.4)	161 (62.9)	
Improved	774 (14.1)	608 (13.9)	79 (13.4)	42 (16.7)	45 (17.6)	
Stamina						< 0.001
Worsened	1035 (18.9)	803 (18.4)	155 (26.4)	29 (11.5)	48 (18.8)	
No change	3761 (68.8)	3025 (69.3)	373 (63.4)	189 (75.0)	174 (68.0)	
Improved	667 (12.2)	539 (12.3)	60 (10.2)	34 (13.5)	34 (13.3)	

## Discussion

E-cigarette use is associated with numerous oral health consequences, including, but not limited to, xerostomia, oral candidiasis, oral mucosal lesions, halitosis, dental caries, and periodontal disease [9, 12, 19, 38]. Although the negative impact of tobacco use on oral health can, to a degree, be mitigated by good oral health behaviors, quitting smoking, and not resorting to E-cigarette use, is the most effective way to ensure good and sustained oral health [22–24]. In this context, dental students and professionals are considered role models for the community and should be at the forefront of fighting dental diseases and the associated deleterious habits. The present survey aimed to assess self-reported oral hygiene practices and the perceived effects of E-cigarettes among dental students across 11 different countries. Collectively, the results revealed good oral hygiene practices including tooth brushing and using additional oral hygiene means, but it also revealed that more E-cigarette users reported dry mouth and heart palpitation compared to non-smokers. Unexpectedly, however, E-cigarette users reported some improvements in some of their physiological functions compared to tobacco smokers or non-smokers.

Owing to a lack of studies on the oral health effects amongst dental students who are E-cigarette users, we weighed the results of our study against what has been reported in the general population. Many recent population-based studies in USA found an association between E-cigarette use and/or dual use, and untreated dental caries [39], self-reported bad oral health [40], chronic obstructive pulmonary disease [41] and other respiratory [42] and health symptoms [43]. Although a recent systematic review stated that E-cigarette use was less harmful than smoking conventional cigarettes, it pointed to a greater susceptibility of E-cigarette users to the development of alterations in oral soft tissues than ex-smokers and non-smokers, and concluded that neither the safety nor efficacy of E-cigarette use in the long-term, either as a smoking cessation aid or as an alternative to tobacco smoking that is less harmful to health, has been established [44].

The present findings concur with previous studies among dental students elsewhere [26, 45]. However, dental students still seem to need some motivation in the use additional oral hygiene devices such as flossing, mouth rinses, and/or interdental brush use and even fluoride-containing paste. Dental students, the future dental professional, through good oral hygiene attitude and practices, can act as role models and play a positive role in improving the oral health status of their patients and the community at large [26, 46].

Another important finding of the present study was the significantly higher proportion of E-cigarette users who had dry mouth and black tongue when compared to non-smokers. This finding is not surprising, as E-cigarette use has been reported to cause numerous oral mucosal conditions such as xerostomia and hairy tongue [9, 12, 19]. These findings agree with those of previous studies that reported a significant association between E-cigarette and oral mucosal diseases [11, 22, 38]. The harmful consequences of nicotine on oral tissues have been well established [11, 38]. Thus it is important to include information about the harmful effects of E-cigarettes in the oral health/dental education curriculum, and to train personnel on how to support quitting the habit [32]. Additionally, the dental curriculum and that of other health disciplines should include information on the oral/general health impact of E-cigarettes [32, 47].

More E-cigarette users reported better physiological functions, especially breathing, mood, taste, physical status, and memory, compared to non-smokers and tobacco users. Although these findings are consistent with many previous studies that reported improvement of general health outcomes, they must be interpreted with caution since such improvement were in context of switching from traditional smoking to E-cigarette use [2, 19, 34, 48]. In other words, after long-term exposure to the very deleterious effects of conventional smoking, the switchers perceive the less deleterious effects of E-cigarettes as an improvement.

The main strength of the present study is the large sample size and involvement of students from various countries with different economic and cultural backgrounds. Nevertheless, the study has some limitations that should be considered when interpreting the results. The main limitation is the self-reported nature of responses, which might have introduced bias. But this might not be of such great concern given that the respondents are dental students: that is to say they can easily determine whether there are alterations in their mouths, and whether these alterations, if present, may be E-cigarette-induced. This is also applied to the self-reported DMFT. Another limitation is the non-response bias, and thus the results cannot be extrapolated to non-participants. Also, despite the multicultural, multi-county sample, the percentage of E-cigarette users in the total sample was relatively small, and subsequently we were not able to run multivariate analysis so as to identify predictors of the outcomes. It follows that the results of the study should be interpreted with caution. Finally, we did not investigate the time of cigarette smoking before switching to E-cigarette use among E-cigarette users with previous experience of cigarette smoking. We focused only on the current status of smoking whether cigarette smoking, E-cigarette use, dual smoking or never.

Overall, the perceived improvement in health outcomes and the wrong perception about the safety of E-cigarettes may explain the growing popularity of E-cigarettes [5]. Unfortunately, E-cigarette users do not view E-cigarette use (vaping) as a harmful habit, and studies have shown that vaping is considered favorably by users concerning overall health perception [49–51]. This emphasizes the urgent need for educating dental students about the long-term health effects of E-cigarettes and implementing tobacco cessation programs.

## Conclusion

Irrespective of the noted good oral hygiene practices among dental students, those who were E-cigarette users reported more oral health-related conditions, particularly xerostomia and black tongue, and heart palpitation, even though they unexpectedly reported significant improvement in their physiological functions compared to non-smokers.

## Data Availability

The datasets supporting the findings of this article are available from the corresponding author.

## Abbreviations

E-cigarette: Electronic cigarettes
FDA: Food and drug administration
IL: Interleukin
TNF: Tumor necrosis factor
IFN: Interferon
DMFT: Decayed, missing, filled teeth
SPSS: Statistical package for the social sciences
US: United States
